# Systematic Review and Clinical Insights: The Role of the Ketogenic Diet in Managing Glioblastoma in Cancer Neuroscience

**DOI:** 10.3390/jpm14090929

**Published:** 2024-08-31

**Authors:** Jose Valerio, Matteo Borro, Elisa Proietti, Livia Pisciotta, Immanuel O. Olarinde, Maria Fernandez Gomez, Andres Mauricio Alvarez Pinzon

**Affiliations:** 1 Neurosurgery Oncology Center of Excellence, Neurosurgery Department, Miami Neuroscience Center at Larkin, South Miami, FL 33143, USA; 2 Internal Medicine Unit, Department of Internal Medicine, IRCCS Ospedale Policlinico San Martino, Largo R. Benzi 10, 16132 Genova, Italy; borromatteo@libero.it; 3 Department of Internal Medicine (DIMI), University of Genova, Viale Benedetto XV, 6, 16132 Genova, Italy; 4 Operative Unit of Dietetics and Clinical Nutrition, Department of Internal Medicine, IRCCS Ospedale Policlinico San Martino, Largo R. Benzi 10, 16132 Genova, Italy; 5 Neurosurgery Department, Latino America Valerio Foundation, Weston, FL 33331, USA; 6 MCIFAU Cancer Center of Excellence, Memorial Cancer Institute, Memorial Healthcare System, Hollywood, FL 33021, USA; 7 Cancer Neuroscience Program, The Institute of Neuroscience of Castilla y León (INCYL), Universidad de Salamanca, 37007 Salamanca, Spain; 8 Institute for Human Health and Disease Intervention, Division of Research, FAU Charles E. Schmidt College of Medicine, Florida Atlantic University, Boca Raton, FL 33431, USA

**Keywords:** glioma, ketogenic diet, ketosis, fasting

## Abstract

Recent scientific research has shown that the ketogenic diet may have potential benefits in a variety of medical fields, which has led to the diet receiving a substantial amount of attention. Clinical and experimental research on brain tumors has shown that the ketogenic diet has a satisfactory safety profile. This safety profile has been established in a variety of applications, including the management of obesity and the treatment of drug-resistant epileptic cases. However, in human studies, the impact of ketogenic therapy on the growth of tumors and the life expectancy of patients has not provided results that are well characterized. Consequently, our purpose is to improve the comprehension of these features by succinctly presenting the developments and conclusions that have been gained from the most recent study that pertains to this non-pharmacological technique. According to the findings of our study, patients with brain tumors who stick to a ketogenic diet are more likely to experience improved survival rates. However, it is required to conduct additional research on humans in order to more accurately define the anti-tumor efficiency of this diet as well as the underlying processes that support the therapeutic effects of this dieting regimen.

## 1. Introduction

Glioblastoma (GBM) is the most common primary central nervous system (CNS) tumor, affecting diverse age groups, with an incidence rate of 5.81 per 100,000 individuals. The frequency of occurrence in the elderly is three times higher than in young children. Glioma, a type of tumor in the central nervous system, is prevalent in 29% to 35% of adolescents and young adults aged 15 to 39. The incidence rate is 3.41 per 100,000 individuals [1,2]. Due to its high mortality and impact on quality of life, glioma represents a global problem with a crucial need for improvement in treatment techniques and therapies.

Treatment efficacy varies significantly with age: Children with high-grade gliomas (HGGs) have poor prognosis, with frequently limited long-term survival ranging from months to a few years after diagnosis [3,4], whereas pediatric patients with low-grade gliomas (LGG) have good overall survival (OS) [5,6] despite significant tumor- and treatment-related morbidity [7]. Malignant transformation makes the prognosis less favorable in adults with low-grade gliomas [8,9].

It is currently possible to treat GBM by a variety of approaches, including chemotherapy, radiation treatment, tyrosine kinase inhibitors, monoclonal antibodies, and surgical procedures. On the other hand, it is essential to keep in mind that each of these prospective treatment techniques is associated with a substantial amount of risks and the possibility of undesirable outcomes [1]

Throughout the years, there has been an increasing interest in supplementary and alternative therapies. This interest has been made possible by the considerations listed above.

As of late, there has been a growing interest in the scientific community about the utilization of ketogenic diets (KDs) as a complementary treatment alongside conventional therapy for the treatment of cancer, particularly in the case of cancers that affect the central nervous system (CNS). The explanation for this is that KD targets the metabolic makeup of cancer cells in a specific manner.

### 1.1. Ketogenic Diet

The ketogenic diet (KD) is originally known to be a first-line non-pharmacological treatment for drug-resistant epilepsy in children, but its uses currently vary [10]. The classical KD is a nutritional approach characterized by its necessarily low-carbohydrate content, higher lipids, and normoprotein composition involving a ketogenic ratio of 4:1 or 3:1, i.e., 4 and 3 g of lipids for every gram of carbohydrates and protein, respectively [11]. However, there are several diets defined as ketogenic in which the proportion of macronutrients can change. Other models considered ketogenic are, for example, the modified Atkins diet, the one with the integration of medium-chain triglycerides, and the low-glycemic index one [12].

Under normal physiological conditions, carbohydrates are dismantled into glucose monomers, which are then used by the brain as the fuel of choice. During prolonged fasting or in the absence of this energy substrate, the liver mobilizes fat reserves, increasing fatty-acid oxidation [10]. The large quantities of acetyl-CoA produced by beta-oxidation cannot enter the Krebs cycle. Thus, they are sent to the production of ketone bodies [13].

A condition of systemic ketosis is induced in the body by the use of the ketogenic diet (KD), which functions by imitating the physiological consequences of fasting. This condition can be verified by determining whether or not ketone bodies are present in either the blood or the urine [14]. Ketone molecules, comprising ß-hydroxybutyrate, acetone, and acetoacetate, have the capability to cross the blood–brain barrier and function as alternative sources of energy, thereby serving as a substitute for glucose [14].

### 1.2. Metabolic Changes of Neoplastic Cells

The metabolic mechanisms of tumors justify the new lines of research focusing on exploring the effectiveness of certain nutritional approaches, especially those that are low in glucose or involve intermittent fasting [15,16]. A unique characteristic that is frequently seen in tumoral cells, particularly malignant glioma, is an abnormality in the system that processes energy. Because of this aberrant metabolism, there is a possibility that the frequency with which the tumor acquires new mutations may grow. Because of the Warburg effect, tumor disorders display a specific metabolic phenotype. This phenotype is characterized by a shift in energy metabolism toward aerobic glycolysis. Although oxygen is present, anaerobic glycolysis emerges as the predominant source of energy for cells. Following glycolysis, pyruvate undergoes fermentation, which results in the formation of lactate [17]. In terms of further modifications, such as the increased production of lactate, it would suggest that metastasis has begun to occur [18]. The extensive reorganization of cellular metabolic systems is the result of the metabolic alteration that was brought about. This process involves redirecting the normal function of mitochondria to generate reactive oxygen species (ROS), carbon skeletons, and other beneficial compounds that promote the formation and proliferation of tumors. In addition, glycolysis and other metabolic pathways inside the cell are disrupted, which leads to the facilitation of unregulated cell proliferation, resistance to programmed cell death, invasion of local and distant tissues, and loss of cellular specialization [19]. Because it ensures the availability of a variety of energy substrates, such as ATP during hypoxia and carbon sources that enable proliferation, the Warburg effect makes it easier for tumors to proliferate. For oncologists, the detection of these abnormalities, such as the aberrant activation of glucose-fermentation pathways, serves as crucial markers that enable them to make diagnoses [17]. In addition to utilizing glucose metabolism, neoplastic cells not only exploit the amino acid metabolism route in an aberrant manner, but they also employ glucose metabolism. To be more specific, it has been shown that tumor cells make use of glutamine, serine, leucine, and aspartate. The nucleotides and proteins that are favorable for tumor cells are created from these amino acids, which act as substrates to produce these molecules [19,20].

In addition, tumor cells depend heavily on aerobic glycolysis and mitochondrial substrate-level phosphorylation (mSLP) for ATP production due to the inefficiencies of oxidative phosphorylation [21]. Aerobic glycolysis rapidly breaks down glucose to ATP and serine necessary for tumor growth and proliferation. mSLP involves succinyl CoA ligase-catalyzed production of ATP during the conversion of succinyl CoA to succinate. Succinyl CoA is derived from glutaminolysis that metabolizes glutamine to glutamate, then α-ketoglutarate, which is subsequently converted to succinyl CoA. The amino group removed from glutamate to synthesize α-ketoglutarate is added to oxaloacetate by aspartate aminotransferase to form aspartate. Therefore, glucose and glutamine are the major substrates from which ATP, serine, and aspartate, which are necessary for tumor survival, are derived.

### 1.3. Theories Regarding Carcinogenesis: A Focus on Mitochondria and Relation to KD

Two main scientific theories attempt to explain the phenomenon of carcinogenesis. The somatic mutation theory of cancer predicts that mutations in tumor-suppressor genes and proto-oncogenes are recognized as the main culprits of the unregulated growth of tumor cells. In fact, according to this theory, cancer is known as a genetic disease [22]. The mitochondrial metabolic theory, on the other hand, proposes that cancer is best explained as a metabolic, hence nongenetic, disease in which proliferating tumor cells cannot survive or grow without the carbon and nitrogen needed for the synthesis of metabolites and ATP [23]. The latter theory seems to define the phenomenon more accurately, and although cancer cells may originate from different tissues or genomic abnormalities, they are mostly dependent on fermentation metabolism via glycolysis and glutaminolysis pathways, offering a potentially non-toxic therapeutic target [24].

The Metabolic Mitochondrial Theory would seem to be the most accredited, also because it would be able to provide an explanation for the oncogenic paradox [25] that was first introduced by Albert Szent-Gyorgyi to describe a seemingly contradictory phenomenon in cancer biology [26]. This paradox refers to the fact that a specific malignant transformation, leading to cancer formation, can be triggered by a wide range of nonspecific events [26]. So, despite the diversity of these triggers, they can all lead to the same outcome: the transformation of normal cells into cancer cells. This apparent contradiction has presented a significant challenge to researchers trying to understand the common mechanisms underlying carcinogenesis [27]. Researchers believe they have solved the oncogenic paradox by identifying a single pathophysiological mechanism for the origin of cancer, namely the prolonged loss of oxidative phosphorylation, following damage to the mitochondria. Although the various triggers of cancer are many and diverse, these factors all cause a loss of mitochondrial function and, therefore, an inability to produce energy in the cells [28].

The ketogenic diet, high in fat and low in carbohydrates, forces the body to use lipid substrates as the main source of energy, producing ketones. These are usable by healthy cells as an alternative fuel but not easily usable by tumor cells, in which the mitochondria are damaged [24]. Therefore, the ketogenic diet, especially when integrated in the “press–pulse” approach where dietary therapy represents the “press” and the glutamine antagonism is the “pulse” with glutamine antagonist 6-diazo-5-oxo-L-norleucine [29], which can be seen as a strategy to exploit the metabolic vulnerability of cancer cells by targeting their altered energy mechanism described by the oncogenic paradox [28].

### 1.4. Mechanism

The ketogenic diet has been suggested as a possible therapeutic method for the treatment of GBM, with the potential for its anti-tumor actions to target both intracellular and extracellular components [30].

Research that was conducted in living creatures (in vivo), as well as in laboratory conditions (in vitro), has shown that glioma cells are highly dependent on glucose as a source of energy. Because of this, these cells go through a state of energy deprivation if there is an absence of glucose. In contrast, glioma cells do not possess the metabolic capacity to adjust to fasting and make use of ketone bodies as an alternative source of energy [31]. Healthy brain cells are able to modify their metabolism to accommodate fasting. This would cause metabolic stress in tumor cells, making them more vulnerable to treatment and possibly increasing the efficacy of conventional treatments like chemotherapy and radiotherapy. As a result, this would indicate the necessity for additional research to be conducted in the clinical setting [14].

Insulin modulation is another consequence that has been suggested to be caused by the ketogenic diet, which is related to intracellular pathways. The ketogenic diet is responsible for a reduction in blood sugar levels, which in turn leads to a reduction in the activation of the Akt/mTOR and Ras/MAPK signaling pathways. These pathways are driven by insulin and are thought to play a role in the development of cancer [32,33]. In addition, it has been discovered that the ketogenic diet can change insulin-related signaling molecules, such as C-peptide or IGF-1, which have been associated with an increased risk of cancer [17].

Apoptosis is triggered in mouse models of astrocytomas when glucose restriction is present, and the ketogenic diet is responsible for activating the AMPK sensor, which is responsible for apoptosis. Consequently, this contributes to the preservation of neural cells that are in good health [34].

The action of the ketogenic diet is not limited to glycometabolic regulation alone, since the ketone bodies released can themselves be considered metabolites with neuroprotective action and to counteract oxidative stress. In favor of healthy brain tissue, the ketogenic diet could probably improve the protection of non-diseased parenchyma of the central nervous system from malignant transformation and attenuate the cytotoxic side effects of conventional treatments [35]. It is hypothesized that part of these effects occurs on behalf of a modification of microRNA expression promoted by the ketogenic diet [36].

Among all ketone bodies, it is beta-hydroxybutyrate that carries out the most interesting actions. Its effect is twofold: on one hand, it appears to protect healthy cells by easily transforming into Acetyl-CoA, allowing it to physiologically enter the Krebs cycle. On the other hand, it can hinder the metabolic functioning of unhealthy cells because impaired mitochondrial function prevents the formed Acetyl-CoA from entering the Krebs cycle and producing energy. Consequently, this metabolite may be redirected towards lipogenesis and cholesterol formation in neoplastic cells [30].

Another intracellular mechanism worthy of mention of ketone bodies concerns the action they exert on monocarboxylate transporters. These transporters vehicle molecules such as lactate, pyruvate, and ketone bodies across cells through a competition mechanism. As part of the ketogenic diet, they can therefore regulate both the access of ketone bodies to the cellular environment and the extrusion of lactate. Secondly, it is possible that the ketogenic diet may cause an increase in the amount of lactate that is present in tumor cells, which will impede their clearance and have an effect on the growth and survival of the tumor [37].

Currently, research is being conducted to determine whether or whether the ketogenic diet can influence pyruvate kinase, an enzyme that plays a vital role in glycolysis, as an intracellular target in malignant tumors. To facilitate the conversion of phosphoenolpyruvate and ATP into pyruvate and ATP, pyruvate kinase is a necessary enzyme. This enzyme can be found in four distinct isoforms (27 separate forms).

A phenomenon that is generally known as the Warburg effect is characterized by changed cells exhibiting a tendency to activate the glycolysis and lactate production pathways while simultaneously reducing oxygen intake. This phenomenon has been seen in the past. It has been established that the M2 isoform of the phosphokinase PKM2 plays a significant role in the promotion of the Warburg effect as well as cancer. To be more specific, it makes the production of lactate easier, and it slows the process of oxidative phosphorylation that occurs in mitochondria [38,39].

Although the mechanism underlying this functioning is still poorly understood, PKM2 is believed to exert its pro-oncogenetic actions through the activation of hypoxia-inducible factor 1 HIF-1, constituting a consequent metabolic advantage for tumor cells [40].

According to Ji et al., the ketogenic diet showed in in vitro culture systems of GBM-infected cells the reduction of the activity of PKM2, the Glut1 transporter and other key enzymes in the process of glycolysis. From this, an energy crisis of the neoplastic cells could develop, due to the strict dependence of the cells on the glycolysis pathway [41], also demonstrated by a reduction in the production of ATP by them in situations of ketosis. Without ATP, cell proliferation is inhibited, and apoptosis of tumor cells is activated [31].

Furthermore, according to mouse models of GBM, the ketogenic diet would act on HIF-1, reducing the expression of the receptor, thus inhibiting neoangiogenesis and influencing tumor metabolism [42].

According to Lussier et al., on their study conducted on murine models, the ketogenic diet could be considered an “immune adjuvant”, so much so that it can be used in combinatorial approaches. In fact, it would inhibit tumor growth and proinflammatory environmental conditions, promoting the survival of mice [43].

### 1.5. Ketogenic Diet and Epigenetic Targets

The modulation of epigenetic variables is the subject of yet another theory that was just recently uncovered. This theory suggests that the ketogenic diet might have a positive impact on malignancies. The ketogenic diet has been discovered to have both direct and indirect effects on the genome and the expression of genes, according to Boison and colleagues’ findings. This indicates that it can exert a positive influence on the expression of oncogenes and tumor suppressors during the course of the disease. The authors [44], who indicate that the KD has a direct inhibitory effect on DNA methylation, state that this effect is achieved via raising the levels of adenosine. Furthermore, it has an indirect impact on the modification of histones through a variety of processes, including acetylation, ubiquitylation, methylation, phosphorylation, and beta-hydroxybutyrylation of the lysine component.

Another key component of the ketogenic diet that is highly intriguing is its role in the epigenetic control of GBM and other types of cancer, particularly in relation to microRNAs [45]. This is an exciting aspect of the diet. On the other hand, these are non-coding RNA molecules that have the ability to create associations with messenger RNA sequences that are complementary to one another, which can lead to the suppression or destruction of gene translation [45]. Alterations in the expression of microRNAs are brought about by the tumor, which results in a reduction in tumor suppressors and an increase in oncogenes [46]. It has been demonstrated through research conducted on mice models with GBM that the ketogenic diet can effectively slow down the growth of tumors by modifying the expression of miRNAs in the genetic code. This led to an increase in the effectiveness of anti-cancer therapy, as well as an improvement in the survival rates of patients involved. When compared to mice that were fed a regular diet, mice that were fed a ketogenic diet showed a drop in the expression of chromatin-modifying genes. These genes are known to play a significant role in the invasion of tumors and resistance to chemotherapy. Furthermore, there was an increase in the synthesis of microRNAs that promote the suppression of tumors [47].

### 1.6. Preclinical Studies

Between the years 2000 and the present day, there has been a growing interest in studying the role that combination dietary therapies, such as the ketogenic diet, intermittent fasting, calorie restriction, and fasting, play in the treatment of specific brain cancers during the course of the study. There was a general decrease in the growth of tumors and an improvement in survival rates, according to the findings of in vivo investigations that were conducted on animal models [48,49]. These findings were generally satisfactory.

Despite this, the ketogenic diet has not always been the only focus of the exclusive dietary strategy, which has not always produced the desired outcomes. It was found that an exclusive method, which was established with a ketogenic ratio of 3:1, did not produce any favorable effects on the progression of the tumor or the longevity of mice that were infected with GBM [50,51]. Regardless of whether the ketogenic ratio is increased alone or in conjunction with radiotherapy, the survival rate of animal models is enhanced by increasing the ketogenic ratio of the diet to 4:1 or even 6:1 [52,53]. This is the case irrespective of the method and extent of the ketogenic ratio’s increase. Furthermore, the ketogenic diet, in conjunction with the synergistic action of the medication, particularly bevacizumab, results in an increase in survival, a decrease in the growth of the neoplasm, and an increase in the amount of ATP available for tumor cells [51].

An additional preclinical investigation was conducted with high-grade glioma mouse models, which provided evidence in support of this assertion. In contrast to a standard diet, the study illustrated how the ketogenic diet increased the survival rate of the rodents. Furthermore, studies revealed that tumor cells do not seem to be capable of utilizing ketone bodies. This was illustrated by the fact that sick tissue accumulated a significant quantity of ketone bodies, in contrast to healthy tissue, which contained only trace amounts of these substances [54].

Interestingly, a subsequent investigation conducted on rodents that were adhering to a ketogenic diet produced data that contradicted these conclusions. The study revealed that rodents affected by gliomas were capable of oxidizing ketone bodies and overexpressing monocarbolysate transporter 1. This discovery challenges the hypothesis that brain tumors adhere to a well-defined metabolic pattern [55] and underscores the importance of undertaking further, more comprehensive research. The ketogenic diet was found to be somewhat effective in preclinical research conducted on animal models, as evidenced by meta-analyses [30]. Nevertheless, these studies did not exclusively focus on brain tumors. The aim of the present study is to provide a comprehensive review of hypotheses’ mechanisms and published clinical studies on the role of KD in the treatment of gliomas. Although a systematic review on this subject was recently published by Sargaco et al., we included more previous studies earlier than 2005 and discussed ongoing clinical trials, making our review more comprehensive [12].

## 2. Materials and Methods

We carried out this systematic review according to PRISMA guidelines [56].

We searched for ((glioma) OR (GBM)) AND (ketogenic diet) on PubMed and EMBASE. We uploaded the search results to Rayyan, a platform for sorting search results for systematic reviews and meta-analyses.

After removing duplicate records, we screened the titles and abstracts of the remaining records for the following exclusion criteria:

- Preclinical studies done on animals
- Letters or responses to the editor
- Review articles
- Studies not reporting feasibility and survival

We included all case reports or series, single-arm pilot studies, cohort studies, and randomized controlled trials looking at the clinical feasibility or outcome of KD—alone or in combination with other treatments—in patients with malignant glioma at any disease stage. We considered the following inclusion criteria:

- Original articles;
- Studies with KD as an intervention alone or in combination with other conventional and nonconventional treatments for malignant glioma;
- All published articles to date regardless of age, gender, ethnicity, and country study conducted.

We collected data on the type of study, study design, number of patients, survival data, and diet-related adverse effects. The Jamovi v2.3 program was utilized to compute the median overall survival, which is converted to months.

The website www.clinicaltrial.gov (accessed on 23 August 2024) was then searched, with the same keywords as mentioned above, for ongoing and future studies on the topic and reported after the results of the systematic review.

## 3. Results

**Figure 1:**
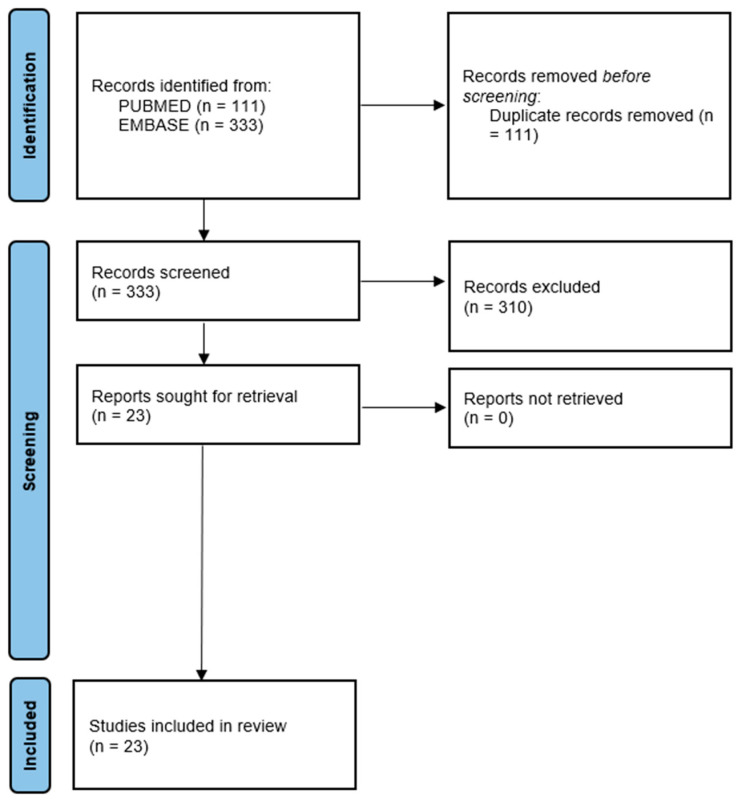
Paper-selection process for this systematic review.

A total of 23 articles were selected for the study after the features of each individual publication were assessed and duplicate papers were excluded. Figure 1 shows the flowchart of the systematic review.

Table 1 outlines all the studies, including 13 prospective studies, three retrospective studies or analyses, five case reports, and two case series.

Afterward, the results are presented in the form of a narrative review, with the goal of incorporating all the progress that has been made over the years.

### 3.1. Published Materials

The initial documented instance of the utilization of KD in the treatment of glioma dates back to 1995. The case report detailed the outcomes of an 8-week regimen that included 60% medium-chain triglyceride oil. Two young female patients with advanced-stage malignant astrocytoma lesions that were unresectable were administered the diet. During that time, the patients had received standard treatment. The results of the Positron Emission Tomography performed on the patients indicated a significant reduction of 21.8% in glucose absorption at the tumor site in both individuals. During the research, one of the patients experienced a substantial improvement in her clinical condition, which prompted her to decide to extend the KD by an additional year. She was not afflicted by any further progression of the disease during this time frame [57], which encompasses the period in question.

In 2010, Zuccoli and colleagues published a paper that examined a 65-year-old woman who had been diagnosed with GBM multiforme. The woman was administered a restricted 4:1 ketogenic diet (i.e., a diet that is high in fat and low in carbohydrates and protein) and water-only therapeutic fasting both prior to and during her treatment. The patient was diagnosed with hypermethylation of the MGMT gene promoter. Corticosteroid medication was not administered. However, supplements containing minerals and vitamins were provided. A significant decrease in body weight of approximately 20% was observed after the treatment was concluded for a 2-month period. Conversely, there was no discernible brain tumor tissue in either the PET or MRI imaging [58].

Han et al. conducted a first-person-perspective study in 2014 on individuals who had been diagnosed with cerebral GBM multiforme. The trial was not randomized and did not include double-blinding. The experimental group consisted of 11 patients who were administered calorie restriction, psychotherapy, and hybaroxia in conjunction with chemotherapy. Conversely, the standard treatment group comprised 23 patients who underwent resections and radio-chemotherapy. The study’s findings demonstrated a statistically significant increase in the average survival time of patients in the experimental group, which was 38 ± 13 months, compared to the conventional group, which had an average survival time of 20 ± 12 months (*p* < 0.05) [59].

Conversely, Schwartz et al. published a case report in 2015 that provided a comprehensive account of the treatment of two GBM patients who were administered KD as their sole form of treatment. We were regretful to advise that both patients exhibited signs of tumor growth [60].

Due to the increasing interest in this co-approach, from 2007 to 2010, a Phase 1 open-label, prospective, single-arm pilot study was performed on 20 patients with recurrent GBM and treated with a mild ketogenic diet to assess changes in quality of life and survival. Patients underwent a ketogenic diet with restricted carbohydrate intake to 60 g/day and a yoghurt drink and oil supplement. No calorie restriction was applied. Every week patients self-monitored urine ketones and wrote a nutritional plan. After 6–8 weeks, or after signs of clinical progression, magnetic resonance imaging was performed to assess disease status. Patients could continue diet treatment alone when the disease was found to be stable or improved or could go for a combination treatment of diet and salvage therapy if in progression. Only after further progression did the diet need to be dismissed. The ketogenic diet was safe and, overall, well tolerated with no serious adverse events related to it being recorded. Results showed a median time to the progression of 5 weeks. Three patients had stable disease at the 6-week follow-up visit that lasted until the 11th, 12th, and 13th week. Overall, results did not permit a satisfied estimation of efficacy, but median overall survival was found at 32 weeks, and a trend for longer PFS was observed in patients with stable ketosis. Anyway, authors speculated that the limited number of participants, the absence of randomization and the lack of a control group, and the failure to achieve significantly lower glucose levels (especially because of concomitant steroid therapy) could represent a bias, thus impeding a good estimation of the efficacy of the KD diet [51].

In 2014, Champ et al. performed a retrospective review of patients with Grade III–IV glioma treated with maximally feasible tumor resection followed by chemoradiotherapy and adjuvant chemotherapy, as well as concomitant KD. Of a total of 134 patients treated at the center, six patients adhered to KD as an add-on therapy. The KD result was well tolerated in all patients with no Grade III or higher toxicity. Results showed that four patients were alive at a median follow-up of 14 months, and the time to recurrence or progression was 10.3 months [61].

In 2017, Santos et al. [62] performed a 3-month randomized study in order to evaluate both toxicity and the therapeutic efficacy of intranasal perillyl alcohol administered in combination with a ketogenic diet regimen for the treatment of patients with recurrent GBM. Thirty-two patients were enrolled and randomized into two different groups: the ketogenic diet group and the standard diet group. Seventeen patients completed the study, nine in the ketogenic diet group and eight in the standard diet group. A partial response was found in 77.8% and 25% of patients in the ketogenic diet group and in the standard diet group, respectively. Progressive disease was observed in 11,1% of the ketogenic diet group and 50% of the standard diet group. Imaging evaluation showed that tumor size was significantly reduced in the ketogenic diet group at 90 days compared with the baseline (*p* = 0.035), in contrast with results found in the control group (*p* = 0.687). The authors concluded that perillyl alcohol may represent a viable option as an adjunct therapy for recurrent GBM when administered concomitantly with a ketogenic diet regime.

In 2018, van der Louw et al. performed a prospective study in three children diagnosed with recurrent pontine glioma without other remaining treatment options. To induce ketosis, the KD was started as a full liquid formula with a 4:1 diet ratio and used for a maximum of 2 weeks. When ketone levels of 3 mmol/L were reached, the liquid formula was modified into a standard KD (range diet ratio 1.5:1–2.0:1) with strictly calculated and prepared meals. The safety profile reported by the authors was good with no severe adverse events. Overall, the survival rate was 16.5, 6.4, and 18.7 months, respectively [63].

In 2018, Martin–McGill et al. conducted a study to assess the practicality and acceptability of a modified ketogenic diet in individuals diagnosed with glioma. A total of six male patients were recruited for a trial lasting 3 months. The modified ketogenic diet (KD) was defined by a nutritional composition consisting of 70% fat and a restriction of dietary carbohydrates to 20 g per day, which accounted for 3–5% of the total energy requirements. The concept of fasting was not understood. Four participants successfully concluded the study and attained a state of ketosis. No significant adverse events were seen, and the diet was well tolerated. A questionnaire was administered during the study to assess the level of patient interest in participating in a larger clinical trial. The results indicated a significant level of interest in such a procedure among glioma patients [64].

In 2019, van der Louw et al. evaluated the feasibility and safety of KD during standard treatment of chemoradiation in patients with GBM multiforme. The protocol comprehended a full liquid KD to induce ketosis within 2 weeks before the start of chemoradiation, followed by solid food and medium-chain-triglyceride emulsions for 6 weeks to maintain ketosis. Of the 11 patients enrolled, six were able to complete the study. No severe adverse event was reported, and overall survival ranged between 9.8 and 19.0 months. The authors concluded that KD was feasible and safe as an adjuvant therapy to standard chemoradiation [65].

From 2017 to 2019, a prospective, non-blinded, randomized, pilot study was performed by McGill et al. in patients newly diagnosed with GB (WHO grade IV) who had a recent surgical resection or biopsy and received, or were approaching, radiotherapy or chemotherapy NCT03075514. Twelve participants were randomized into two different diet groups for a 12-month follow-up period: the modified ketogenic diet (MKD) and the medium chai triglyceride ketogenic diet (MCT). The difference between the two groups consists in the amount of fats: the MKD and MCT are 80% fat and 5% carbohydrate and 75% fat and 10% carbohydrate, respectively. The aim of the study was to determine which diet is most deliverable and best adhered to by patients to test clinical effectiveness as a primary outcome (overall survival) in future definitive trials. Results showed a lower retention rate than expected: in fact, out of 12 patients who enrolled, only 10 patients started dietary intervention, and only four met the primary endpoint of the 3-month dietary intervention. The main reason for such poor retention was found in the periodic finding of low urinary and blood ketones that was defined as “demoralizing”, thus leading us to think that the diet was not working. The evaluation of Quality of Life for those patients who continued for 12 months showed that global health status improved in the MDK group and worsened in the MCT group. But, during interviews, both groups reported a fantastic quality of life and described the diet as offering a sense of “control” whilst receiving their tumor treatment. In their conclusion, the authors hypothesized that the effectiveness of this dietary intervention could be evaluated with a shorter period of adherence, like a 6-week period [67].

In 2019, a retrospective study performed by Woodhouse et al. [66] examined the feasibility of attaining ketosis and the safety of the modified Atkins diet in patients undergoing standard treatment (temozolomide 75 mg/m2 and 30 fractions of radiation therapy to a total dose of 59.4 Gy for a total of 6 weeks) for glioma. Twenty-nine patients with Grades III–IV astrocytoma who previously followed such a scheme, with a follow-up period of 4 weeks after radiation, were included in the analysis. The authors distinguished between pseudoprogression, such as the radiographic evidence of an inflammatory response to standard treatment, which is hypothesized to be linked to a survival benefit in GBM and tumor progression. Results showed that all patients were able to achieve ketosis without Grade 3 or 4 adverse events, but nutritional ketosis was maintained by only 79% of patients. Pseudoprogression was found in 55% of patients, whereas tumor progression was found in 17% of patients. By extrapolating data from GBM-only patients, pseudoprogression was found in 58% of them and tumor progression in 21%. Twenty-one percent of GBM patients showed stable disease or regression. The authors evaluated also the overall survival after 2 years, finding a 26.7% overall survival for GBM patients, which was considered in line with the current literature. The authors concluded that further studies were needed to understand the true efficacy of KD as an add-on therapy and the correct “dose” of serum ketosis that is required to obtain ketosis in the brain.

In 2020, Voss et al. [70] performed a prospective case-control clinical study in 50 patients diagnosed with recurrent malignant gliomas treated with a calorically restricted ketogenic diet combined with intermittent fasting or a standard diet, in addition to re-irradiation. The primary endpoint was the evaluation of the progression-free survival rate at 6 months, and the secondary endpoints were the overall survival (OS), frequency of epileptic seizures, rate of ketosis, and quality of life of enrolled patients. The diet scheme included 3 days of KD (21–23 kcal/kg/d), followed by 3 days of fasting and, again, 3 days of KD. Eight patients quit before or during the study. The fasting ketogenic diet group had 20 participants, whereas the standard diet group had 22 participants. Results related to the primary endpoint showed no significant difference in both the progression-free survival rate at 6 months (20% in the fasting-ketogenic diet group vs. 16% in the standard group, *p* = 0.713) and the median progression-free survival (75 days in the fasting-ketogenic diet group and 91 days in the standard diet group, *p* = 0.729). In January 2019, 43 patients out of 50 enrolled were deceased. Overall, survival showed no statistically significant difference among the two groups (median overall survival in the fasting-ketogenic diet group of 331 days vs. 291 days in the standard diet group, *p* = 0.978). By selecting only the patients in the fasting-ketogenic diet group with a glucose level below the median glucose level of all patients enrolled, the authors found that this population presented both a significantly longer progression-free survival (median PFS 111 days) and a significantly longer overall survival (median OS 348 days). The evaluation of adverse events showed similar numbers among the two groups. The fasting-ketogenic diet group reported one focal status epilepticus and some mild adverse events, including headache and nausea. The authors concluded that the lack of efficacy of the dietary intervention could be related to the small population size, short treatment schedule consisting of only 5 radiation fractions, the presence of different prevalences of pre-treatments in the two different groups (i.e., bevacizumab in the dietary group and surgical resection in the control group), and a possible, more metabolic flexibility of glioma cells. Finally, they focused on metabolic changes hypothesizing that lower levels of glucose and higher levels of blood ketosis are mandatory to achieve effectiveness for further studies.

From 2013 to 2021, a prospective, randomized, open-label study NCT01865162 was performed to evaluate the efficacy, safety, and tolerability of a 4:1 ketogenic diet administered adjunctively to standard radiation and temozolomide chemotherapy in patients with Refractory/End-stage Astrocytoma Grade 4. From 2014 to 2022, an analogous Phase 1 study was started by the same authors, exploring the efficacy and safety of KD administered adjunctively to standard radiation and temozolomide chemotherapy in patients with early GBM NCT02302235. The evaluation and intervention protocols of the two studies were identical, and results were reported together because of the slow recruitment rate [68]. All patients were treated for 6 months with a 4:1 (fat/protein + carbohydrate) ratio by weight diet, with 10 g CH/day, 1600 kcal/day. Group 1 consisted of newly diagnosed patients, and Group 2 consisted of patients already treated with radiation and temozolomide or recurrent glioma. Blood glucose and ketone levels, in both blood and urine, were regularly tested. Eight patients were enrolled overall: Four subjects were in Groups 1 and 4 in Group 2. KD was well tolerated, and no adverse events related to dietary intervention were found, other than mild or moderate levels. Among enrolled patients, seven of them died of GBM and only one was currently in KD at the time of publication of the results. The mean survival time from diet initiation to death was 20 months and 12.8 months, for Group 1 (range 9.5–27) and Group 2 (range 6.3–19.9 months), respectively. The mean survival from the time of diagnosis till death was 21.8 months (range 11–29.2) and 25.4 (range 13.9–38.7) for Group 1 and Group 2, respectively. The long survival found in Group 2 was unusual and attributed to two patients that had secondary GBM developed from Grade 3 astrocytoma, whose survival from diagnosis was 19 and 28.7 months. The mean time from diet initiation to MRI progression was 3.4 months and 3.9 months for Group 1 and Group 2, respectively. The authors concluded that the main result of the study was that a prolonged (6 months) diet program with a rigorous, 4:1 ketogenic diet has been well tolerated when delivered as a total meal replacement schedule using standardized recipes and ready-made meals [68].

Moreover, the authors focused on the difficulty for patients to adhere to KD because of lack of palatability, difficulty in meal preparation, and lack of standardization. Previous studies found that KD could be tolerated only for 6–12 weeks [51,62,79]. They pointed out that a possible solution could be to provide the diet as a complete ready-made meal-delivery program, identical for all patients. With these adjustments, authors showed a good adherence to the protocol with five out of eight patients who completed it. Efficacy was not evaluated due to the poor sample size.

In 2020, Panhans et al. published a retrospective case series 32,508,561 in which they evaluated the role of KD in CNS malignancies in 12 patients identified between 2015 and 2018. Included malignancies were GBM, astrocytoma, or oligodendroglioma. Patients were required to follow a 3:1 ketogenic diet for 120 days. All patients with available blood-test data reached ketosis during the 120 days after starting, and all patients were adherent. Eight patients kept on with the diet or a low-carb, modified version of it on their own beyond the 120th day of the study. At the time of publication, two patients were deceased because of a progressive disease, and 10 patients presented a stable disease. All patients presented a certain degree of weight loss and decreased BMI during KD, but they reported an overall improvement in symptoms, including increased energy levels, mobility, mood, and cognitive function. Moreover, the authors found that in four patients, there was an improvement in imaging evaluation, suggestive of radiographic response. Some patients who experienced seizure as a cancer symptom presented an easier control over it with the establishment of KD [69].

Another single-arm study NCT02286167 was performed from 2014 to 2019 by Schreck et al. on adult patients diagnosed with GBM who had completed over 80% of prescribed concurrent radiation therapy and adjuvant temozolomide without Grade 3 or 4 toxicity. The diet proposed was the modified Atkins diet that is designed to provide a more palatable, less restrictive, but effective alternative to the strict KD, particularly for adults. Twenty-five patients with biopsy-confirmed World Health Organization Grade 2-to-4 astrocytoma were enrolled in an 8-week intervention. The weekly diet consisted of 2 fasting days (calories < 20% calculated upon the estimated needs), followed by 5 modified Atkins diet days (net carbohydrates ≤ 20 g/d). The study demonstrated that this KD scheme is safe in patients with glioma and feasible, with a good adherence (over 72%). Moreover, the results of the study showed a production of quantifiable systemic and cerebral ketosis in participants, thus demonstrating that a systemically delivered dietary intervention can generate quantifiable changes in cerebral metabolite [71].

In 2021, Perez et al. retrospectively analyzed the adherence, safety, and overall survival of patients diagnosed with pontine glioma who underwent KD. They found five patients to be included, with all of them that adhered to diet for over 3 months. Tolerance of the diet was good, with only mild gastro-intestinal complaints, one borderline hypoglycemia, and one hyperketosis. The median overall survival was 18.7 months, range 9 months–30 months. The authors concluded that KD was well tolerated, and there was no evidence of a negative impact on clinical outcomes. Specifically, the overall survival rate, found to be higher than the generally reported 11 months, was hypothesized related to a potential bias, small sample size, and the presence of a long-term survivor [72].

Interestingly, in 2021, Seyfried et al. [73] reported of a 26-year-old patient treated exclusively with a low-carbohydrate ketogenic diet consisting mostly of saturated fats and minimal vegetables with promising results. The patient was diagnosed with IDH-mutant GBM (WHO Grade 4) but refused to undergo standard treatment. He adhered to the ketogenic diet regimen and achieved glucose/ketone index values near 2.0 or below within 2 weeks. Serial imaging analysis showed evidence of interval slow contrast-enhanced tumor progression, so the patients agreed to awake debulking craniotomy after almost 3 years. The histological analysis confirmed the previous diagnosis. The patient kept on with a strict ketogenic diet regimen in the first period after surgery but then, because of the evidence of stable disease, relaxed, keeping a glucose/ketone index ranging from 5 to 10. One year after surgery, imaging showed interval progression of the lesion. The patient thus restarted with improved adherence to the diet, and various imaging performed within 2 years showed continued slow interval progression of the disease without the formation of vasogenic edema. The authors report that at the time of the report (April 2021), the patient was in good physical condition and presented a good quality of life, except for occasional seizures. The authors hypothesized that such a long survival (80 months at the time) could be related to the presence of IDH1 (R132H) mutation, which presents favorable survival, the young age of the patient, and the overall good adherence to the diet with low levels of blood glucose.

In 2021, a single-institution Phase I clinical trial was performed by Porper et al. [74] in 13 newly diagnosed and recurrent gliomas. Patients were randomized into three cohorts, all receiving radiotherapy: modified Atkins diet alone or in combination with low-dose or high-dose metformin therapy. Six patients had a newly diagnosed disease and seven patients had a recurrent disease. Within 4 weeks, 5 of 13 patients enrolled, all coming from the cohort with the lower metformin dosage and KD, discontinued diet regimen. Reported adverse events were nausea, asymptomatic hyperuricemia, anorexia, and difficulty in keeping dietary goals. Results of the trial showed a median progression-free survival of 10 months for a newly diagnosed disease, 4 months for a recurrent disease and a median overall survival of 21 months and 8 months, respectively. The authors showed some limitations of the study, including the short duration of the metabolic intervention (8 weeks), the small size of the trial, and the absence of caloric restriction. Moreover, the authors suggested for future trials not to exceed the metformin dosage of 850 mg BID.

A prospective, randomized study on patients with recurrent GBM was performed from 2013 to 2019 NCT01754350 by Voss et al. Fifty patients were enrolled and treated with re-irradiation. Patients were randomized into two different groups: standard diet and a combination of a calorie-restricted ketogenic diet and intermittent fasting. Both groups received professional counseling by a dietician. The ketogenic-fasting intervention was characterized by two calorically restricted intervals separated by 3 days of fasting. Calory restriction was 21–23 kcal/kg/d and carbohydrate intake was limited to 50 g/d; fasting was from Day 4 to Day 6 with an unlimited intake of fluids. Results showed no statistically significant difference in progression-free survival among the two groups. A possible interpretation of this result was that both the intervention group and the control group had lower calorie intake, thus representing a confounding factor [75].

In 2022, Phillips et al. performed a prospective case series in which they investigated whether a combined metabolic approach among fasting and KD could be feasible, safe, and effective on GBM patients. Ten patients were recruited at different stages of tumor progression and treated with a 5–7-day fasting diet every 1–2 months, combined with a modified KD during intervals to keep glucose ketone index under 6. The results of the study showed that a mean of 161 ± 74 days of the combined approach was reached, including 34 ± 18 (21%) days of prolonged fasting. The glucose ketone index was under the cutoff limit both during fasting and during the interval period. Body weight was reduced by a mean of 11.2% from the baseline, and the most common adverse events reported and attributed to the strategy were fatigue, irritability, and feeling lightheaded. No interferences were noted with standard oncological treatments. The authors concluded that such a strategy was feasible and safe. However, the effectiveness, with a median survival of 13 months, was encouraging but not reasonably true because the study was biased by the presence of diseases in different stages due to half of the participants having a poor prognosis at the time of inclusion, a lack of overlap with standard treatment in order to enhance stress to pathologic cells, corticosteroid exposure that contrasts the aims of KD, and fasting [76].

In 2022, Schwartz et al. [77] reported results of a long-term follow-up of patients diagnosed with aggressive primary brain tumors treated with a 6-week adjuvant KD combined with standard therapy (radiation and temozolomide) after initial neurosurgery. Twelve patients were enrolled, two patients were enrolled with the previous study in which KD was started after failure of other therapies, and 10 patients were enrolled with the described protocol; one patient did not complete the protocol. The two patients enrolled with the previous protocol died of GBM. Nine patients completed the protocol, and three of them were in a progression-free stage at 80, 64, and 62 months since diagnosis (all young, two of them with the IDH-1 mutation and one with a Grade III astrocytoma). The other six patients, who were older, had a disease progression in 8,11, 9, 6, 6, and 6 months after diagnosis. The authors concluded that KD was possibly working against GBM only in younger patients. Thus, it was suggested to present such an approach only in a specific age range.

Recently, in 2024, the same group of authors reported a 64-year-old woman with isocitrate dehydrogenase (IDH)-wildtype GBM who underwent the standard treatment protocol together with an intensive, multimodal KD program for 3 years. Specifically, after completing surgical resection, radiation therapy and temozolomide were administered and then followed by six cycles of adjuvant temozolomide. Meanwhile, the patient was prescribed with a diet protocol comprehending prolonged fasting, time-restricted feeding, and a modified KD with the intention to maximize the tolerability and efficacy of the standard treatment. Specifically, prolonged fasting consisted of eight 7-day, fluid-only fasts, with the first starting 4 days before chemoradiation, the second occurring in the third week of treatment, and the latter six starting 4 days before each temozolomide cycle. On all other days, a time-restricted KD with only two meals a day was prescribed. All meals were composed of 60% fat, 30% protein, 5% fiber, and 5% net carbohydrate by weight and comprised whole foods. During the first 2 years of such a scheme, the patient showed clinical improvement with no signs of cancer progression on imaging. In the 3rd year, due to concomitant extra-disease reasons, adherence to the protocol was reduced, the patient experienced clinical decline, and signs of progression were found. The clinical situation worsened rapidly, and the patient then chose the best supportive care. Adverse events were registered, mostly related to fasting with fatigue, diarrhea, and cold intolerance. Despite the significant commitment required to adhere to the program, the authors reported a positive effect attributed to diet behavior, including reduced chronic neck, back, and bilateral shoulder pain. Moreover, the authors hypothesized a synergic mechanism between standard treatment and the metabolic program, suggesting an enhancement of the sensitivity of tumor cells to the standard treatments by restricting their access to glucose and growth factors. The authors then affirmed that a possible explanation of the long survival of the patient could be, at least partially, related to a certain number of positive prognostic features of the clinical situation, including gender, complete resection, and borderline methylation status [78].

### 3.2. Ongoing Trials

This section provides a condensed summary of all presently published studies, including detailed information on primary and secondary outcome measures. We identified a total of 18 clinical trials on the clinicaltrials.gov website utilizing both “glioma” and “ketogenic diet” as keywords. The findings of the seven trials that were conducted were previously addressed in the section preceding this one. Nevertheless, the analysis did not include one of the trials, as it was not included in the seven trials because it had been withdrawn. Nine unique trials constitute the subsequent listing.

The NCT03278249 study aims to evaluate the capability of a modified Atkins diet in inducing ketosis in patients over 18 years of age diagnosed with Grade III or IV malignant glioma. The study is characterized by the presence of a single group of assignments. The primary outcome is the achievement of ketosis measured by serum beta-hydroxybuterate within 6 weeks. The secondary outcome comprehends both the measurement of progression-free survival in 6 months and overall survival within 2 years [80].

The NCT02939378 study is an open-label, non-randomized case-control study with the purpose of evaluating the safety and efficacy of KD as an adjuvant therapy to chemotherapy for recurrent GBM, as previously evaluated in the pilot study described above 33292598. Inclusion criteria comprehend a Karnovsky Performance Score of at least 60 points, and histological confirmation of the diagnosis of GBM multiforme was documented with a recurrence or progression after resection/debulking, radiation, and temozolomide chemotherapy. Patients are divided into two groups: experimental ketogenic diet group, with the aim of keeping ketone levels more than 2 mmol/L during chemotherapy, and the standard diet group. The primary outcome is the evaluation of the onset of adverse events during KD. Secondary aims focus on evaluating the effects of combination therapy on tumor size by MRI, overall survival, blood ketosis with urine and blood tests, and the quality of life measured by the Karnofsky Performance Scale [81].

A pilot study with the purpose of evaluating the tolerability and effects of concomitant treatment with metformin and KD in patients diagnosed with high-grade glioma is currently in the recruitment phase (NCT04691960). The study started in 2016, and primary completion is estimated to be at the end of 2024. A nutritionist will help participants to prepare their own meals. The diet is characterized by a 3:1 fat-to-carbohydrate + protein ratio for 5 days that is increased to 4:1 if the patient fails to achieve ketosis measured by urine ketosis of 1.5 mmol/L or 27.0 mg/dL. Fasting would be added lastly if no ketosis is reached. Thirty millilitres per day of Medium Chain Triglycerides are added to the daily diet to enhance ketosis. Metformin is administered 850 mg/day at Week 8 and titrated up to 850 mg BID at Week 10 and, subsequently, TID at Week 12 if tolerated. Outcomes comprehend the proportion of patients who achieve and maintain a ketogenic status and the proportion of patients who can tolerate metformin treatment during ketosis [82].

Another pilot, open-label observational study NCT01535911 was active but not yet recruiting; it will evaluate the effects of an energy-restricted KD in the treatment of newly diagnosed GBM, in addition to radiation therapy and standard-of-care chemotherapy. The purpose of the study is to evaluate if the diet can decrease the size of the cancer or prevent its growth. Total calories consumed by each subject will be targeted to 20-to-25 kcal/kg/day, and the evaluation of ketosis is made on a twice-daily measurement [83].

An interesting Phase 2, randomized, two-armed, multi-site study of 170 patients with newly diagnosed GBM multiforme is currently in recruitment NCT05708352. Enrolled patients will be randomized into two groups: KD or standard anti-cancer diet. Both groups will receive the standard-of-care treatment for GBM. The KD will last for 18 weeks with daily ketone and glucose-level evaluation. The primary endpoint is the evaluation of the overall survival at Month 18 in order to support the hypothesis that KD can be beneficial in newly diagnosed GBM. Secondary endpoints include the evaluation of changes in the quality of life assessed with Functional Assessment of Cancer Therapy—Brain score and Functional Assessment of Chronic Illness Therapy—fatigue, changes in progression-free survival, differences in the cognitive performance measured by Hopkins Verbal Test and Trail Marking Test A/B survey scores, changes in physical activity evaluated with the modified Godin leisure questionnaire survey score, and the Fitbit [84].

Another two-stage, two-cohort, open-label Phase 2 trial, NCT05183204, is currently recruiting to evaluate the safety and effects of the treatment with paxalisib, a new blood–brain penetrant PI3K/mTOR inhibitor, and metformin during KD in patients with newly diagnosed GBM. Arm 1 consists of patients diagnosed with MGMT unmethylated GBM, and Arm 2 consists of recurrent GBM patients, regardless of methylation status. Both groups will undergo KD throughout the trial and receive paxalisib at 45 mg/day before being titrated to 60 mg/day after 28 days, and metformin from the first cycle was received at 850 mg/day then uptitrated to 850 mg TID at Cycle 3. The primary endpoint is the evaluation of progression-free survival, defined as the survival rate at 6 months, measured by the occurrence of a progression event or death due to any cause prior to 6 months. Secondary endpoints consist of the evaluation of overall survival, defined as the time of the first study treatment to death from any cause, modification in insulin levels, and changes in tumor glucose uptake values, measured by FDG-PET/DCE MRI [85].

The NCT05564949 study, focusing on the efficacy of KD in extending the survival of patients with high-grade gliomas and brain metastases, is currently in the recruitment phase. The study design consists of a classic KD for 3 months with a possible subsequent extension upon compliance evaluation. In order to compare the outcome measurements, a historical control group is used. Patients measure their urine ketosis with urine test strips and capillary ketones with blood ketone meters daily, and they complete a diet diary from the start until the end of the study. The primary outcome evaluated is overall survival, from inclusion into the study until death from any cause, assessed up to 36 months. The secondary outcome includes changes in brain tumor size evaluated with MRI, time to progression, assessment of quality of life using the FACT-BR (version 4) survey, and changes in functional impairment evaluated with the Karnofsky Performance Scale [86].

A single-arm, prospective cohort, Phase 1 study evaluating a 4-month KD in combination with standard-of-care radiation and temozolomide for patients with newly/recently diagnosed GBM, NCT03451799 is currently active. A dietitian creates personalized meal plans for each patient to induce and keep metabolic ketosis. The primary outcome is focused on safety, designed as both the proportion of participants experiencing a 10% decrease in weight or body mass index and reducing their BMI less than 18.5 within 1 month from the beginning, as well as the number of KD-related adverse events. Secondary outcomes comprehend the evaluation of adherence, such as the proportion of patients able to maintain blood ketone levels over 0.3 mM/L for over 50% of days on the study starting 2 weeks after the initiation of the ketogenic diet. Overall, survival, time to progression, quality of life at 2 and 4 months, and cognitive function were evaluated with different scales [87].

The NCT04730869 study, performed by Matthew CL Phillips et al., provided the encouraging findings of preliminary data shown by the same authors [76] and is currently recruiting. The study is an open-label, single-arm study focused on determining whether using KD together with standard oncological treatment is feasible, safe, and effective in GBM patients compared to previous trials. The diet intervention consists of a modified ketogenic diet characterized by one or two 1 h eating windows per day, allowing oils, meats, vegetables, nuts, seeds, limited berries, and a multivitamin between fasting periods, such as two 5-days fasts during chemoradiation therapy, followed by a 5-day fast during each adjuvant chemotherapy cycle. The primary endpoint is the measurement of the proportion of patients that can sustain functional ketosis (defined as a mean daily blood glucose-to-ketone ratio of ≤6) during chemoradiation at Week 9. The secondary outcome includes the same goal as the primary outcome, but at Weeks 24 and 33, changes in body weight at Week 33 and the appearance of adverse events (of any grade) change in performance status as measured by the Eastern Cooperative Oncology Group Performance Status scale; changes in leisure/exercise activity are measured by the Godin Leisure–Time Exercise questionnaire, and changes in quality of life are measured by the Functional Assessment of Cancer Therapy—Brain questionnaire, as well as the evaluation of progression-free survival and overall survival [88].

## 4. Discussion

We have documented all case reports, case series, and clinical studies that concentrate on the administration of a ketogenic diet as part of the therapy of patients diagnosed with glioma, regardless of its grade. A growing interest in this approach in human beings has been sparked by the promising results observed in animal models, where the establishment of a ketogenic environment was associated with a more effective growth suppression in brain tumors and, occasionally, a prolonged survival timespan [89].

Despite the presence of a tendency towards the usefulness of KD in the treatment of GBM, which is expressed in terms of feasibility and safety in almost all papers included in the reviews, clear evidence for its use in clinical practice is still very limited. In 2017, a systematic review by Winter et al. [14] showed that results from preliminary studies were suggesting safety and feasibility of KD in gliomas. A potential benefit of such a treatment was already hypothesized at that time, but lots of limitations came out. Particularly, the small sample size of each study, the heterogeneity of analyzed studies, the lack of control groups, and the placebo effects, together with the expectancy bias due to the forced choice to go for open-label trials because of the impossibility of hiding dietary modifications, came out as limitations in the evaluation of its practical effectiveness.

Among the studies included in our review, overall survival varies significantly, ranging from 8 month to 60 months, but this heterogeneity can be influenced by several characteristics such as age, disease stage, adherence to therapy, treatment efficacy, tumor location, and specific tumor characteristics. Standard treatment, such as chemoradiation performed after surgical resection, followed by adjuvant temozolomide chemotherapy, is associated with a median survival of 12–15 months [90]. We found that the overall survival that results from our analysis is 16.0 months.

The specific survival found in each study reported in our review can be found in Table 1.

Despite the presence of some reports of reduced survival as compared to the known median survival time of standard therapy in GBM, most of the studies included present an overall survival that is improved. This is in line with previously published results [91].

Of note, all the clinical trials reported include standard of care as the major treatment of gliomas. Research mainly focuses on KD as an add-on therapy in order to take advantage of the synergic mechanism with chemotherapy and radiotherapy [92]. Acidosis and low blood glucose levels contrast cancer growth. Synergism between chemotherapy and KD has been shown in several cancers, including breast cancer [93] and gastric advanced cancer [94]. Research in pancreatic cancer in murine models showed that KD, although without direct effects on tumor growth, was able to triple the survival benefits [95] by increasing radiation sensitivity. Interestingly, radiotherapy and KD seem to present a similar behavior as shown in a murine model of lung cancer. Different concentrations of glucose and beta-hydroxybutyrate, administered to emulate KD, showed the capability to enhance the anti-tumor effect of radiotherapy by reducing glucose and increasing the energy-supply ratio from fat. Beneficial effects of such an association were found in head and neck cancers [96].

Regarding adverse events and tolerability, all studies with available data concluded that KD can be used safely without emerging severe symptoms or complications.

Table 2 summarizes all signs and symptoms related to dietary intervention reported by each author.

No Grade III or IV adverse events were reported, except for a Grade III neutropenia that authors labeled as only “possibly related” to KD [71]. Of note, and interestingly, the patient described by Phillips et al. reported an overall positive effect of the ketogenic metabolic therapy that reduced chronic pain and induced a feeling of well-being [78]. Comparable positive effects were reported also by Panhans et al. with an overall improvement in symptoms such as mobility, mood, and cognitive functions [69]. Analogous results were found in pre-clinicals but also in clinical studies [51,68].

In contrast, the strict dietary adherence of patients needed to achieve an optimal glucose/ketone index can be challenging in patients diagnosed with cancer. As reported by Klein et al. [68], one possible solution to the problem can be the delivery of ready-made meals. Specifically, their dietary program consisted of five meals/day (breakfast, morning snack, lunch, afternoon snack, dinner), varied each day on a 2-week cycle. Meals were prepared by a catering facility and delivered frozen each week.

Due to the high frailty and malnutrition of cancer patients, the ketogenic diet may not always be considered a viable option, especially from an ethical point of view. This would explain why studies on GBM in humans are decidedly inferior to preclinical ones, not only due to concerns that may be linked to the tolerability of the ketogenic regime and ketosis but also on the quality of life and, more generally, on the well-being of patient suffering from cancer [66]. As emerged from the first study, conducted on pediatric patients, food intake, quantified by dietary history, even improved during the ketogenic diet phase, satisfying the nutritional and energy needs of the enrolled subjects. This demonstrated that the ketogenic diet does not necessarily affect the nutritional status of the subjects [57] Moreover, regarding the quality of life, Martin–McGill et al. found an improvement on the part of the subjects involved through the administration of validated questionnaires [67]. An important argument about quality of life in this particular disease has been conducted by Sargaço et al. [12]: the level of quality of life of each patient is strictly related to factors other than KD, such as the stage of the disease, general well-being, symptoms of the disease and of adverse events coming from chemoradiotherapy, and the patient’s response to therapy. For these reasons, they concluded that the evaluation of quality of life does not target the contribution of KD to that achievement, good or bad. Probably, a better variable to evaluate could be the adherence of each patient to the prescribed diet.

Taking together all these findings, the ketogenic diet could prove to be a valid non-pharmacological approach in the treatment of brain tumors. Analyzing the rationale under this statement for the two main distinct mechanisms, it has been suggested that fasting, which involves a series of metabolic alterations considered unfavorable for tumor cells, and ketosis, a metabolic situation that involves the use of alternative energy sources, and ketone bodies, which reduce the “classic” feed for neoplastic cells (i.e., glucose), may act as a synergic mechanism with chemotherapy [76]. Moreover, ketone bodies can perform neuroprotective functions, and in the context of tumor diseases, they could represent an advantage for healthy cells. It is not clear whether both mechanisms can be useful simultaneously for the treatment of cancer, nor whether one of the two is more effective than the other. It is also true that dietary regimes formulated on the basis of caloric restriction, which has always been more studied, have shown notable and promising effects in animal models [97], supporting the hypothesis that fasting may be the primary mechanism responsible for “anti-tumor” action. The ketogenic diet is certainly less studied, and the need for improvements in its application brings out new challenges for researchers, including the establishment of a standardized dietary protocol. However, according to some studies, the higher ketogenic ratio of the diet, i.e., the achievement of a more intense ketosis, thus characterized by a low glucose ketone index, would seem to be of greater benefit for the treatment of tumors, making normal cells more resistant to stressors (including radiation and chemotherapy) and cancer cells more sensitized [76,98,99,100]. In this context, the low blood glucose levels obtained by strict adherence to KD may play a role even in cancer with normal or upregulated mitochondrial functions by reducing available glucose, thus contrasting tumor growth.

From these bases, the need to create more homogeneous interventions that include a single protocol, with a ketogenic ratio and a caloric quantity that is as homologated as possible, arises. Moreover, long-term effects of KD and specific results related to different categories of patients (age, gender, and ethnicities) will be needed to increase the consistency of results and identify populations more prone to achieve better outcomes (overall survival and progression-free survival). Furthermore, to fully understand the true efficacy of KD, future trials need to comprehend a control group, better with randomization and masking of the dietary intervention. As previously mentioned, masking can be difficult in these patients. A possible suggestion can be to adjust the scheme adopted by Demirel et al. in patients with acute spinal cord injuries. The authors described that, in patients able to swallow, the research kitchen provided liquid or solid meals according to patient needs and randomization [101]. Another important point to discuss and improve in the future is patient adherence to dietary programs, which is mandatory to achieve a state of ketosis. Although previous studies have shown a positive association between a higher ketogenic ratio (higher proportion of fats compared to protein and carbohydrates) of KD and health benefits, such as better seizure control and a reduced risk of developing diabetes [102,103], such a restrictive and strict dietary regimen may result in poor patient compliance [104]. The comparison of macronutrient intake across different KD interventions can aid in refining dietary strategies and enhancing the diet’s overall effectiveness [105]. Moreover, the use of methods to assess adherence has shown some improvements. Li et al. [106] described systematically revised instruments used to assess the adherence to KD. Among subjective methods, the most frequent is the classic diet-adherence-assessment tools, such as a 24 h diet recall and diet records, which may be useful to establish a real-time picture of the subject’s eating habits but may lead to a considerable underestimation of food intake. The most widely used objective measure to assess adherence to KD remains the ketone biomarker, which has the advantage of overcoming the bias of subjective measures [107]. Because of the lack of a gold standard to assess dietary adherence, objective and subjective instruments should be integrated with precise standardized protocols to fulfill that need [108]. Furthermore, in order to increase patient confidence and adherence, it would be extremely useful to to build a customized nutritional recommendation system by harnessing algorithms generated by artificial intelligence to create personalized meal and recipe solutions based on the patient’s medical history, habits, and dietary preferences [109].

Furthermore, the comparison of the efficacy of only KD over the standard of care for GBM is limited because none of the presented clinical trials included a cohort treated with only KD without the standard of care. However, Seyfried et al.’s case report was on a patient with IDH1-mutant GBM treated with only KD, without chemotherapy and radiotherapy, highlighting the potential efficacy of only KD for GBM treatment. The patient’s tumor progression was slowed down by surgical debulking and long-term KD alone. Although the patient had intermittent seizures throughout the course of the disease, he had a better quality of life due to minimum therapy-related adverse effects. This contrasts with the declining quality of life attributed to the adverse effects of chemoradiotherapy. The perceived advantages of KD for GBM may only be apparent in the presence of, or complemented by, IDH1 mutation, which limits the ability of the tumor to resist the negative effects of KD on growth and proliferation [73].

Our search presents some limitations. First, there is a selection bias because of the lack of randomization of the cohort studies. Moreover, the inclusion and comparison of different kinds of studies and reports make it difficult to compare data and extrapolate conclusions. Finally, the lack of blinding in the already-limited randomized studies predisposes to reporting and observer bias.

## 5. Conclusions

KD was demonstrated to be safe and well-tolerated in most individuals who participated in clinical trials. Even though the efficacy of this treatment in humans is still uncertain, clinical trials have shown a beneficial effect. From preclinical research, evidence was obtained that is more consistent and indicates that the use of this treatment as an additional treatment for gliomas, particularly in the reports and clinical trials regarding GBM, could have a beneficial effect. The implementation of standardized glucose/ketone index (GKI) across clinical trials provides a strong basis for comparing the feasibility of KD regardless of the dietary regimen used. Better blinding strategies and the inclusion of a control group to enhance the comprehension of overall survival and progression-free survival are among the most critical new research and translational medicine elements in cancer neuroscience that must be implemented. Finally, a subject for future research that was not investigated in the presented trials is the efficacy of inhibiting glutaminolysis and mSLP pathways in isolation or in combination with KD in treating GBM.

## Figures and Tables

**Table 1:** Summary of included studies.

Study	Type of Study	Number of Participants	Overall Survival
Nebeling et al., 1995 [57]	Case Report	2	60 months and 48 months
Zuccoli et al., 2010 [58]	Case Report	1	NA
Han et al., 2014 [59]	Prospective Study	11	Mean Survival: 38 +/− 13 months
Schwartz et al., 2015 [60]	Case Report	2	NA
Rieger et al., 2014 [51]	Prospective Study	20	Median: 32 weeks
Champ et al., 2014 [61]	Retrospective Analysis	134	Median: 14 months
Santos et al., 2017 [62]	Prospective Randomized Study	37	NA
Van der Louw et al., 2018 [63]	Prospective Study	3	16.5, 6.4 and 18.7 months
Martin-McGill et al., 2018 [64]	Prospective Study	6	NA
Van der Louw et al., 2019 [65]	Prospective Study	11	9.8 and 19.0 months
Woodhouse et al., 2019 [66]	Retrospective Study	29	Not evaluated
Martin-McGill et al., 2020 [67]	Prospective Study	12	Median: 67.3 weeks
Klein et al., 2020 [68]	Prospective Randomized Study	8	Group 1: 20 months (9.5–27) Group 2: 12.8 months (6.3–19.9)
Panhans et al., 2020 [69]	Retrospective Case Series	12	90.8–19.0 Months
Voss et al., 2020 [70]	Prospective Randomized Study	50	KD: 331 days SD: 291 days Low glucose KD: 348 days
Schreck et al., 2021 [71]	Prospective Study	25	NA
Perez et al., 2021 [72]	Retrospective Study	5	Median: 18.7 Months
Seyfried et al., 2021 [73]	Case Report	1	80 Months
Porper et al., 2021 [74]	Prospective Randomized Study	13	21 Months in patients with newly diagnosed disease 8 Months in patients with recurrent disease
Voss et al., 2022 [75]	Prospective Randomized Study	50	250–485 days
Phillips et al., 2022 [76]	Prospective Case Series	10	Median: 13 Months
Schwartz et al., 2022 [77]	Prostective Study	12	Not Reported
Philli s et al., 2024 [78]	Case Report	1	36 Months

**Table 2:** Summarized all reported Signs and Symptoms related to KD.

Study	Adverse Events Related to KD
Nebelin et al., 1995 [57]	No reported symptoms
Zuccoli et al., 2010 [58]	hyperuricemia
Han et al., 2014 [59]	N/A
Schwartz et al., 2015 [60]	No significant adverse events
Rieger et al., 2014 [51]	Weight loss, diarrhea, constipation, hunger
Champ et al., 2014 [61]	Constipation, asthenia, weight loss, nephrolithiasis, hypoglycemia
Santos et al., 2017 [62]	Not reported
Van der Louw et al., 2018 [63]	Hypoglycemia, hyperkeratosis, vomiting, refusal to eat, asthenia, constipation
Martin-McGill et al., 2018 [64]	Constipation
van der Louw et al., 2019 [65]	Constipation, nausea/vomiting, hypercholesterolemia, hypoglycemia, diarrhea, low carnitine concentration
Woodhouse et al., 2019 [66]	Grade 2 constipation occurred in 1 patient. Grade 1 fatigue and nausea probably due to standard therapy
Martin-McGill et al., 2020 [67]	Hypokalemia, hypocalcemia, hypernatremia, hyperkalemia, constipation
Klein et al., 2020 [68]	Weight loss, hunger, nausea, dizziness, asthenia, constipation
Panhans et al., 2020 [69]	Asthenia, weight loss, nausea, vomiting, headache, decreased appetite
Voss et al., 2020 [70]	Epileptic seizures, headache, nausea
Schreck et al., 2021 [71]	Grade 2 adverse events: Leukopenia, nausea, diarrhea, fatigue, or seizure. Grade 3: neutropenia (possibly related)
Perez et al., 2021 [72]	Hypoglycemia, constipation, hyperkeratosis, vomiting asthenia, hyperuricemia
Seyfried et al., 2021 [73]	Not reported
Porper et al., 2021 [74]	Nausea, asymptomatic hyperuricemia, anorexia
Voss et al., 2022 [75]	Gastrointestinal symptoms, headache, muscle cramps
Phillips et al., 2022 [76]	Fatigue, irritability, and feeling lightheaded. No grade 3 or higher adverse events.
Schwartz et al., 2022 [77]	Not reported.
Phillips et al., 2024 [78]	Prolonged fasts caused mild fatigue, diarrhea, and cold intolerance. No adverse events for KD.

## Data Availability

No new data were created or analyzed in this study. Data sharing is not applicable to this article.
